# Effects of Prehabilitation With Advanced Technologies in Patients With Musculoskeletal Diseases Waiting for Surgery: Systematic Review and Meta-Analysis

**DOI:** 10.2196/52943

**Published:** 2024-12-12

**Authors:** Stefania Guida, Jacopo Antonino Vitale, Eva Swinnen, David Beckwée, Silvia Bargeri, Federico Pennestrì, Greta Castellini, Silvia Gianola

**Affiliations:** 1 Unità di Epidemiologia Clinica IRCCS Istituto Ortopedico Galeazzi Milan Italy; 2 Rehabilitation Research Group (RERE) Department of Physiotherapy, Human Physiology and Anatomy Vrije Universiteit Brussel Brussel Belgium; 3 Spine Center Schulthess Klinik Zurich Switzerland; 4 Department of Rehabilitation Sciences and Physiotherapy University of Antwerp Antwerpen Belgium; 5 Direzione Scientifica IRCCS Istituto Ortopedico Galeazzi Milan Italy

**Keywords:** prehabilitation, advanced technologies, musculoskeletal diseases, joint arthroplasty, elective surgery, patient outcomes, mobile health, postoperative function

## Abstract

**Background:**

Prehabilitation delivered with advanced technologies represents a great chance for patients to optimize pre- and postoperative outcomes, reduce costs, and overcome travel-related barriers.

**Objective:**

We aim to evaluate the effects of prehabilitation delivered with advanced technologies on clinically relevant outcomes among patients affected by musculoskeletal diseases and waiting for surgery.

**Methods:**

We searched the PubMed, EMBASE, Cochrane Library, PEDro, and CINAHL databases up to February 2, 2023. ClinicalTrials.gov was also searched for registered protocols. Randomized controlled trials and nonrandomized intervention studies with adult participants of both sexes, affected by any musculoskeletal disease, and undergoing prehabilitation with advanced technologies or standard care were included. Study selection, data extraction, and critical appraisal were conducted in duplicate. Data were pooled for meta-analysis using random-effects models. Certainty of evidence was assessed for the primary outcome with the GRADE (Grading of Recommendations Assessment, Development and Evaluation) system. The primary outcome was function. Secondary outcomes were pain, strength, risk of fall, autonomy in the activities of daily living, patient satisfaction, health-related quality of life, adverse events, and adherence to treatment.

**Results:**

Six studies (7 reports), focusing on patients undergoing total knee or hip arthroplasty and primary meniscal tear and spine surgery were included. We found different prehabilitation programs: mindfulness-based stress reduction, exercise, education, or a combination thereof. Prehabilitation delivered with advanced technologies proved to be more effective in improving function in candidates for knee or hip replacement (Western Ontario McMaster Osteoarthritis Index “function” subscale before surgery: mean difference [MD] –7.45, 95% CI –10.71 to –4.19; *I*^2^=0%; after surgery: MD –7.84, 95% CI –11.80 to –3.88; *I*^2^=75.3%), preoperative pain (MD –1.67, 95% CI –2.50 to –0.48; *I*^2^=0%), risk of fall (MD –2.54, 95% CI –3.62 to –1.46; *I*^2^=0%), and postoperative stiffness (MD –2.00, 95% CI –2.01 to –1.99; *I*^2^=87%). No differences were found in pain 1 month after surgery. Data from studies including participants undergoing primary meniscal tear and spinal surgery could not be pooled.

**Conclusions:**

Prehabilitation delivered with advanced technologies may be better than standard care in improving pre- and postoperative function among candidates for knee or hip arthroplasty. No quantitative results have been achieved on spine surgery candidates or other musculoskeletal diseases.

**Trial Registration:**

PROSPERO CRD42022345811; https://www.crd.york.ac.uk/prospero/display_record.php?RecordID=345811

## Introduction

Musculoskeletal diseases are one of the biggest contributors to global disability [1] and the volume of related surgeries continues to increase worldwide [2]. Highly invasive surgery imposes substantial stress on the body’s physiology [3], affecting not only physical aspects but also physiological, cognitive, and psychosocial factors [3,4]. Undergoing major interventions results in a high systemic inflammatory response and increases catabolism and oxygen demand in the immediate postoperative period [3,5]. Moreover, patients commonly experience preoperative anxiety, sleep disturbance, depression, and pain, which predict higher postoperative disability, poor function, and health-related quality of life [6,7]. Therefore, the waiting period before elective surgery should provide the opportunity for patients to identify and manage these internal contextual factors optimizing their chances of postoperative recovery [5].

The term “prehabilitation” refers to actively preparing patients for surgery through interventions such as exercise, nutritional support, psycho-cognitive training, or a combination thereof, to enhance functional capacity and improve tolerance to upcoming surgical stress [3,5]. Although prehabilitation is highly valued by patients, the evidence supporting its benefits in the musculoskeletal field is varied and uncertain [3,8]. Some studies demonstrate benefits per function, pain, quality of life, and length of hospital stay [8-10], while others show comparable outcomes to no treatment [11-16]. Moreover, prehabilitation is a noninvasive and low-cost intervention [17,18]. Given all these potential benefits, it would be worth recommending [13], especially for patients who are frail or present risk factors for poor recovery [13,19]. However, the evidence from these trials and reviews is relatively weak, often with a high risk of bias (RoB), with critical heterogeneity in populations, interventions, and outcome measures [20]. Additionally, patients’ compliance varies widely [21]. Digital health interventions hold significant potential to enhance the availability of health care services, facilitating better access and adherence to treatment [22,23].

Following orthopedic surgery, telerehabilitation using advanced technology systems has demonstrated comparable effects to standard treatment, often with lower costs, high patient satisfaction, and adherence [24-28]. Advanced technologies in rehabilitation refer to a broad spectrum of devices and software that integrate hardware and software capabilities to support and improve the assessment, training, or recovery processes in patients with physical or cognitive impairments. Common examples include virtual reality, wearable sensors, robotic exoskeletons, and telerehabilitation platforms [29]. These technologies provide personalized, interactive, and data-driven rehabilitation experiences, often making use of real-time feedback, data analytics, and remote connectivity to enhance the efficiency and effectiveness of rehabilitation programs [30]. In particular, the remote delivery of prehabilitation through technological devices or web-based modalities offers the double advantage of lower costs compared to traditional prehabilitation [31-33] and facilitates monitoring of adherence and results. Nevertheless, to the best of our knowledge, no systematic reviews investigated the effects of prehabilitation with advanced technologies (PrAT) before orthopedic surgery yet. In this context, we aim to evaluate the effects, per function, of PrAT compared to standard care to provide patients, clinicians, and stakeholders evidence to better manage the preoperative phase of candidates.

## Methods

### Study Design

This systematic review was conducted following the Cochrane Handbook guidance and it is reported per the PRISMA (Preferred Reporting Items for Systematic Reviews and Meta-Analyses) statement [32] (section S1 in Multimedia Appendix 1). This study protocol was registered on PROSPERO (CRD42022345811). Differences between study protocol and publication are reported in section S1 in Multimedia Appendix 1.

### Eligibility Criteria

We considered eligible randomized controlled trials (RCT) and nonrandomized intervention studies (NRISs) comparing the effects of PrAT (eg, synchronous or asynchronous sessions of physical exercise, education to autonomous exercise execution, or activities suggestion) and standard care or no intervention, in adult patients affected by a musculoskeletal disease waiting for orthopedic surgery. More details on inclusion criteria are reported in section S2 in Multimedia Appendix 1.

### Outcomes

We considered function as the primary outcome, of physical structures integrity, physiology, activity, and participation [34]. If studies used multiple metrics to assess similar constructs, we prioritized the extraction of outcome measures according to existing core outcome sets. Specifically, we chose Outcome Measures in Rheumatology–Osteoarthritis Research Society International [35] core domain set (WOMAC [Western Ontario and McMaster Universities Osteoarthritis Index] function for the primary outcome) for osteoarthritis and Chiarotto et al’s [36] outcome set for lumbar degenerative diseases (Oswestry Disability Index for the primary outcome). We prioritized multiple outcome measures according to the aforementioned core outcome sets post hoc, as this alignment was necessary to reflect the specific population focus of the included studies.

Secondary outcomes were pain, strength, risk of fall, autonomy in the activities of daily living, patient satisfaction, health-related quality of life, adverse events, and adherence to treatment.

### Literature Search

PubMed, EMBASE, Cochrane Library, PEDro, CINAHL, and ClinicalTrial.gov were searched in July 2022 and updated on February 2, 2023, with no further results. Details on the search strategy are reported in section S2 in Multimedia Appendix 1. Titles and abstracts of the records were screened by 2 independent reviewers (S Guida and JAV), and the full texts were obtained if they met the inclusion criteria. We also screened for eligibility for all references from relevant systematic reviews. A third reviewer was involved in case of disagreement (S Gianola). Rayyan [37] software was used to manage this study’s selection phase.

### Data Extraction

Two reviewers (S Guida and JAV) extracted the following data: study generalities, study methods, population characteristics, intervention and comparator characteristics, outcomes, and possible other important variables. As described in the Outcomes section, we prioritized the extraction of multiple outcome measures for similar constructs according to existing core outcome sets (ie, Outcome Measures in Rheumatology–Osteoarthritis Research Society International and Chiarotto et al’s [36] core outcome set). By adhering to these core outcome sets, we ensure that the outcome measures extracted were both relevant and standardized, facilitating a more coherent and comparable synthesis of findings across the included studies.

For interventions, we applied the reporting checklist of the 12-item Template for Intervention Description and Replication (TIDieR) checklist (section S2 in Multimedia Appendix 1) [38].

### Methodological Quality

Two independent authors (S Guida and JAV) assessed the RoB using The Cochrane RoB tool for RCTs [39] and The Newcastle Ottawa Scale (NOS) for NRISs [40]. Discrepancies were solved by discussion. The description of the domains evaluated by RoB and NOS and the rules for judgment are reported in section S2 in Multimedia Appendix 1. For these phases, we used DistillerSR.

### Data Synthesis and Statistics

The treatment effect has been evaluated using mean difference (MD) or standardized mean difference as appropriate. As we supposed a certain degree of clinical heterogeneity between studies (eg, delivered programs of interventions), we opted for random effects models with 95% CIs. We assessed the presence of statistical heterogeneity using the *I*-squared statistic (*I*^2^). When *I*^2^ was higher than 75% we planned to explore the sources of heterogeneity by subgroup or sensitivity analyses, depending on study findings [41,42]. Analyses were done with Review Manager (RevMan5) software (version 5.2; The Cochrane Collaboration) and Stata13 (StataCorp LLC) [43].

Certainty of evidence was assessed for all the meta-analyzed outcomes with the GRADE (Grading of Recommendations Assessment, Development, and Evaluation) system (section S2 in Multimedia Appendix 1).

## Results

### Study Findings

**Figure 1:**
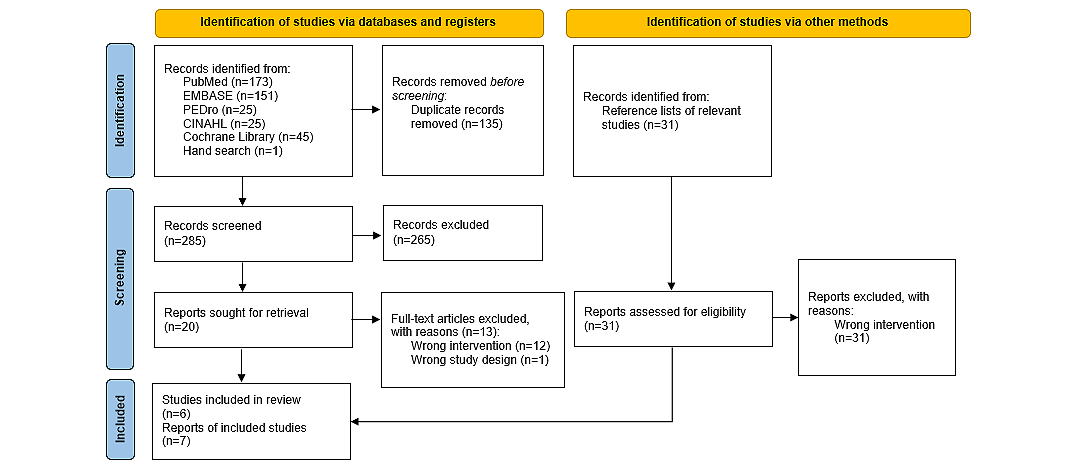
PRISMA flowchart. PRISMA: Preferred Reporting Items for Systematic Reviews and Meta-Analyses.

The searches retrieved 451 records. After removing duplicates, 316 references were screened by titles and abstracts. Twenty studies were considered for eligibility and assessed by full text. Finally, 7 studies were included (Figure 1) [44-50]. Two references belong to the same study, reporting different time points for interested outcomes [45,46].

### Characteristics of the Included Studies

The 7 included studies were published between 2015 and 2021. Four studies were RCTs [44,47-50] and 2 NRISs [45,46]. Most studies were conducted in the United States (n=4) [45-48] followed by Canada (n=2) [44,49] and Korea (n=1) [50]. The main studies’ features are provided in Table 1.

**Table 1:** General characteristics of the included studies.

First author (year)	Study design	Diagnosis	Sample size (N)	Intervention modality	Intervention dosage and characteristics	Weeks (n): (1) of intervention (2) before surgery	Comparator characteristics	Outcomes measures	Assessment time points
Yi et al (2019) [45]	NRIS^a^	Lumbar degenerative disease	48	Asynchronous	Total volume: 960 minutes. Mindfulness-based stress reduction course. 8 audio-video lessons + 6 h audio-video retreat (about 16 h overall)	Not reported (at least 1 session before surgery)	No prehabilitation	MED^b^ per day, VAS-BP^c^, VAS-LP^d^, ODI^e^, EQ-5D-QUALY, EQ-5D-VAS, PROMIS-PF^f^, and PROMIS-PI^g^	Baseline (preoperative visit), 30 days after surgery
Chavez et al (2020) [46]	NRIS	Lumbar degenerative disease	48	Asynchronous	Total volume: 960 minutes. Mindfulness-based stress reduction course. 8 audio-video lessons + 6 h audio-video retreat (about 16 h overall)	Not reported (at least 1 session before surgery)	No prehabilitation	MED per day, VAS-BP, VAS-LP, ODI, EQ-5D-QUALY, EQ-5D-VAS, PROMIS-PF, and PROMIS-PI	Baseline (preoperative visit), 3 months and 12 months after surgery
Yin et al (2015) [47]	RCT^h^	Primary meniscal tear	64	Asynchronous	Total volume: 20 minutes. Web-based tutorial covering anatomy, pathology, and general perioperative instructions	1 week 1-2 weeks	Standard counselling	Ad hoc surveys for patient’s perioperative experience preparedness and satisfaction)	Baseline (preoperative visit), the day of surgery (preoperative), first visit after surgery
Culliton et al (2018) [49]	RCT	Knee osteoarthritis	416	Asynchronous	Total volume: 50 minutes. Digital version of “My Guide to Total Knee Replacement” + 32 educational videos (patient’s expectations on pain, function, restrictions, or exercises) and 2 medical animations of TKA^i^ surgery (about 50 minutes overall).	Not reported (from preadmission visit to surgery)	Paper-printed “My Guide to Total Knee Replacement”	Preoperative expectation survey, KSS^j^, Knee Injury and Osteoarthritis Outcome Score, SF-12^k^, Hospital Anxiety and Depression Scale, Pain Catastrophizing Scale, Social Role Participation Questionnaire, PASS^l^	Baseline (preoperative visit), 6 weeks, 3 months, and 1 year after surgery
Soeters et al (2018) [48]	RCT	Knee or hip osteoarthritis	126	Asynchronous and synchronous	Total volume: 85 minutes. Microsites for information and preparation (with also images and videos) + one face-to-face session	1-2 weeks 2 weeks	One face-to-face session	WOMAC^m^ and length of stay	Baseline (preoperative visit), 4-6 weeks after surgery
An et al (2021) [50]	RCT	Knee osteoarthritis	60	Asynchronous and synchronous	Total volume group A: 900 minutes. Group A: 2-way video call (therapeutic exercise). Group B: Guidebook for exercises and education.	3 weeks 4 weeks	Standard counseling	ROM^n^, PPT^o^, TUG^p^, WOMAC, quadriceps peak torque 60°/s and 180°/s	Baseline (4 weeks before surgery), the day of surgery (preoperative), 6 weeks after surgery
Doiron-Cardin et al (2020) [44]	RCT	Knee or hip osteoarthritis	34	Asynchronous and synchronous	Total volume group A: 3780 minutes. Group A: 2/7 sessions through 2-way video call and 5/7 unsupervised home sessions (therapeutic exercise and education). Group B: 2/7 in-person sessions and 5/7 unsupervised home sessions (therapeutic exercise and education).	12 weeks Not reported	One face-to-face session (home-based)	LEFS^q^, WOMAC, SF-36^r^, TUG, ST^s^, SPW^t^, satisfaction survey	Baseline (preoperative visit), 12 weeks after baseline (preoperative)

^a^NRIS: nonrandomized intervention study.

^b^MED: morphine-equivalent dosing.

^c^VAS-BP: visual analogue scale for back pain.

^d^VAS-LP: visual analogue scale for leg pain.

^e^ODI: Oswestry Disability Index.

^f^PROMIS-PF: Patient-Reported Outcome Measurement Information System Physical Function.

^g^PROMIS-PI: Patient-Reported Outcomes Measurement Information System Pain Interference.

^h^RCT: randomized controlled trial.

^i^TKA: total knee arthroplasty.

^j^KSS: Knee Society Score.

^k^SF-12: Short Form Health Survey.

^l^PASS: patient acceptable symptom state.

^m^WOMAC: Western Ontario and McMaster Universities Osteoarthritis Index.

^n^ROM: range of motion.

^o^PPT: pressure pain threshold.

^p^TUG: timed-up and go.

^q^LEFS: Lower Extremity Functional Scale.

^r^SF-36: Short-Form 36 Health Survey.

^s^ST: stair test.

^t^SPW: self-paced walk.

Overall, 748 participants were considered, ranging from 34 to 416 per study.

Interventions included therapeutic exercise, mindfulness-based exercises for stress reduction, and preoperative information and education. Treatments were mainly focused on (1) online mindfulness-based exercises for stress reduction, including indications for gentle stretching, yoga, and walking (n=2) [45,46]; (2) online educational content, including exercise suggestions (n=3) [47-49]; (3) remotely supervised exercises (n=1) [50]; and (4) remotely supervised and unsupervised exercises plus educational contents (n=1) [44]. The total prehabilitation dosage ranges from 20 to 3780 minutes, delivered over 1 to 12 weeks using an asynchronous (n=4) [45,46,48,49], synchronous (n=1) [50], or mixed (n=1) [44] modality. Comparisons groups were “no intervention” (n=2) [45,46] or “standard care” (n=5; ie, standard counseling, standard education, or one face-to-face session) [44,47-50] as defined by the authors.

As assessed by the TIDieR checklist, all trials reported: complete information on the provided interventions on rationale, setting, and materials; incomplete information on procedures [48], intervention providers [45-48], posology [45,46], tailoring, and planning (all studies). No studies reported information on intervention modification. TIDieR scoring details are provided in Table S1 in section S3 in Multimedia Appendix 1. Most studies reported pain outcomes. Details about outcome reporting are in Table S2 in section S3 in Multimedia Appendix 1.

### Methodological Quality

**Figure 2:**
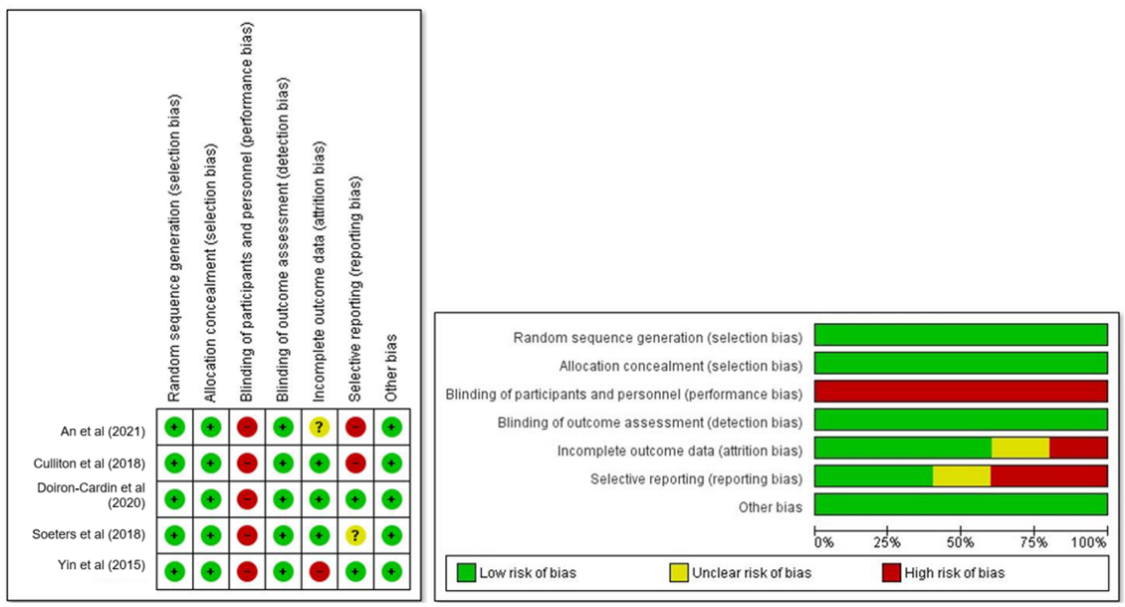
Risk of bias graph and summary.

The RoB assessment of RCTs is reported in Figure 2 [44,47-50]. All studies were judged as having a high RoB. However, the final judgment is impacted by the nature of the investigated intervention that, for its intrinsic features, does not allow blinding of participants and personnel to the delivered treatment. All the RCTs used blinded procedures to randomize and allocate participants and blinded outcomes assessors. Two studies deviated from what the protocol stated per primary outcomes.

The 2 NRISs [45,46] were both assessed with a score of 3/9 of the NOS. The “poor quality” was imputed, for both studies, to a self-reported ascertainment of exposure to the intervention and to the presence of the outcome of interest at the start of the study. One study had too short a follow-up and the other had a high dropout rate. NOS scores are reported in Table 2, with further details in Table S3 in section S3 in Multimedia Appendix 1.

**Table 2:** Newcastle Ottawa Scale quality score summary.

Author and year	Selection (out of 4)	Comparability (out of 2)	Outcome (out of 3)	Overall quality score
Yi et al (2019) [45]	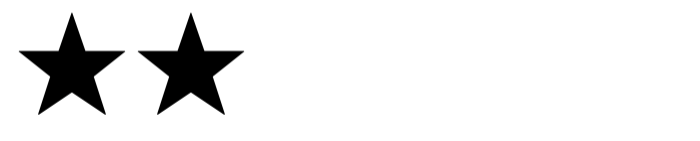		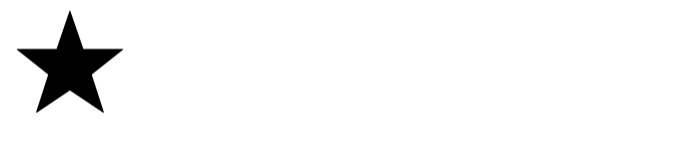	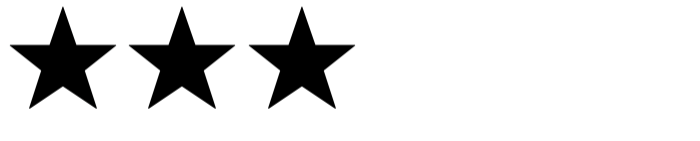
Chavez et al (2020) [46]	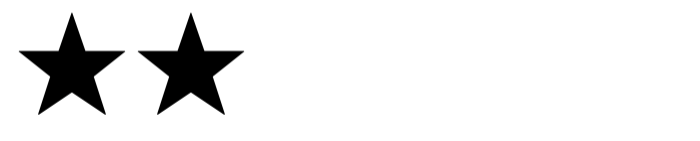		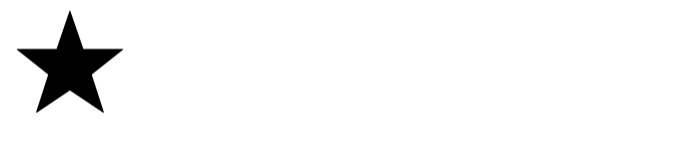	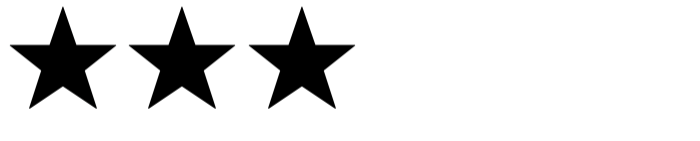

### Data Synthesis on Knee or Hip Prehabilitation

#### Overview

Four studies investigated the effect of PrAT on patients affected by knee or hip osteoarthritis and waiting for arthroplasty.

#### Primary Outcome: Function

**Figure 3:**
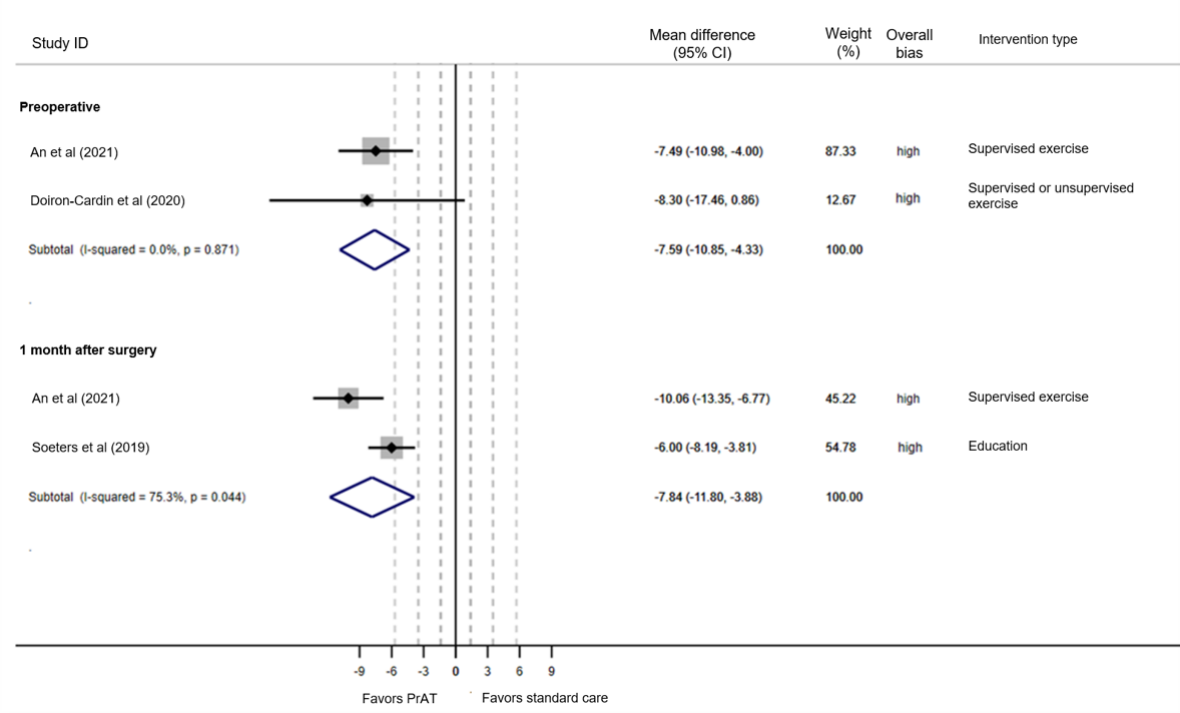
Forest plot WOMAC function subscale. WOMAC: Western Ontario and McMaster Universities Osteoarthritis Index.

Three out of four studies measured the lower limb function using the WOMAC function subscale. With very low certainty of evidence, meta-analyses show a statistically significant better effect of PrAT compared to standard, both at the end of the intervention (2 studies, n=94, MD –7.45, 95% CI –10.71 to –4.19, *I*^2^=0%) and a month after surgery (2 studies, n=186, MD –7.84, 95% CI –11.80 to –3.88, *I*^2^=75.3%; Figure 3 [45,49,51). The main reason for the downgrade was imprecision (clinical effect and sample size; section S2 in Multimedia Appendix 1).

#### Secondary Outcomes

##### Risk of Fall

Meta-analysis of 2 studies (n=94) showed a significant improvement in favor of PrAT on the TUG test at the end of the intervention (MD –2.54, 95% CI –3.62 to –1.46, *I*^2^=0%) compared to standard care, with low certainty of evidence (section S4 in Multimedia Appendix 1).

##### Stiffness

Meta-analysis of two studies (n=186) showed a significant improvement in favor of PrAT on the WOMAC stiffness subscale one month after surgery (MD –2, 95% CI –2.01 to –1.99, *I*^2^=87%), with low certainty of evidence. The forest plot is reported in Figure S2 in section S4 in Multimedia Appendix 1.

##### Pain

Meta-analysis of two studies (n=94) showed a significant improvement in favor of PrAT on the WOMAC pain subscale at the preoperative time point (MD –1.67, 95% CI –2.50 to –0.84, *I*^2^=0%), with very low certainty of evidence. No statistically significant difference in pain was found one month after surgery (2 studies, n=186; standardized mean difference –1.06, 95% CI –2.24 to 0.12, *I*^2^=88%). Forest plots are reported in Figures S3 and S4 in section S4 in Multimedia Appendix 1.

##### Strength

No meta-analysis could be performed on this outcome. Quadriceps strength was investigated by one study [50] showing a statistically significant difference in favor of PrAT, at different time points, for participants undergoing total knee arthroplasty (TKA). Significant differences were observed across pre and postoperative time points (*P*<.001), between groups, as well as a significant time-by-group interaction.

##### Autonomy in the Activities of Daily Living

No meta-analysis could be performed on this outcome. Activity limitations were investigated by one study [49] showing a statistically significant difference in favor of PrAT one year after surgery, for participants undergoing TKA (*P*=.04).

##### Patient’s Experience and Satisfaction

No meta-analysis could be performed on this outcome. One study [49] assessed patient satisfaction and experience reporting no differences between groups. Another study [44] reported high satisfaction with tele-prehabilitation but without any information on the usual care group.

##### Adherence to Treatment

No meta-analysis could be performed on this outcome. Three studies assessed adherence (number of sessions performed versus planned). Two studies [47,48] reported that most PrAT participants completed the intervention (70.83% and 97%, respectively). Another study reported similar adherence rates for both the intervention (between 77% and 73%) and comparison groups (between 80% and 86%) [44].

No studies including participants affected by knee or hip osteoarthritis reported information on possible adverse events related to treatment or PrAT effect on health-related quality of life.

##### Data Synthesis on Other Musculoskeletal Diseases

The effects of PrAT were assessed by 1 study (2 reports) [45,46] on patients affected by lumbar degenerative disease and by 1 study [47] on patients with primary meniscal tear.

#### Primary Outcome: Function

No meta-analysis could be performed on this outcome. One study (2 reports) [45,46], investigating PrAT in participants affected by lumbar degenerative disease, assessed function by the Oswestry Disability Index. No statistically significant differences were found between groups 30 days after surgery (*P*=.12, while better function was found at the 3-month follow-up (*P*=.03).

#### Secondary Outcomes

##### Pain

No meta-analysis could be performed on this outcome. One study (2 reports) [45,46], investigating PrAT in participants affected by lumbar degenerative disease, assessed function by the visual analogue scale for back pain (VAS-BP) and the visual analogue scale for leg pain. VAS-BP was better in the PrAT group 30 days after surgery (*P*=.004). No statistically significant differences were found for VAS-BP and visual analogue scale for leg pain both at the 30 days (*P*=.495) and 3 months (*P*=.06) after surgery time points.

##### Patient’s Experience and Satisfaction

No meta-analysis could be performed on this outcome. One study [47], investigating PrAT in participants with primary meniscal tears, assessed patients’ experience and satisfaction by an ad hoc survey. Participants undergoing PrAT felt significantly more prepared for surgery and satisfied (*P*=.04), and satisfied (*P*=.03) with the received assistance compared to those undergoing standard counseling.

##### Health-Related Quality of Life

No meta-analysis could be performed on this outcome. One study (2 reports) [45,46], investigating PrAT in participants affected by lumbar degenerative disease, assessed quality of life by the EQ-5D-5L questionnaire after surgery. No statistically significant difference between groups was found in both 30 days (*P*=.19) and 3 months (*P*=.27) after surgery.

##### Adherence to Treatment

No meta-analysis could be performed on this outcome. One study (2 reports) [45,46], including participants affected by lumbar degenerative disease, reported an adherence rate to PrAT (number of patients that completed at least 1 of 6 preoperative sessions) of 70.83%.

No studies including participants affected by other musculoskeletal diseases reported information on possible adverse events related to treatment or PrAT effect on strength, risk of falls, and autonomy in the activities of daily living.

## Discussion

### Main Findings

This systematic review aimed to evaluate if PrAT is better than standard care in improving function for patients with musculoskeletal disease and waiting for surgery. We included TKA, total hip arthroplasty, and patients with lumbar degenerative disease undergoing different PrAT interventions per modality (synchronous or asynchronous), prehabilitation main body (exercise, education, instruction, or suggestions), and dosage with a duration of intervention ranging from 1 to 12 weeks. Our analysis suggests that PrAT may increase function, but the evidence is very uncertain at both preoperative (supervised and unsupervised exercises) and postoperative time points (education and exercise) in participants undergoing TKA or total hip arthroplasty. One study assessed the function of spine surgery candidates, with better outcomes 3 months after surgery but not at the 30-day time point. No study assessed the function of other musculoskeletal diseases. The magnitude of the effect for the WOMAC function subscale was critically important for patients undergoing knee or hip arthroplasty. However, the estimated effects were very wide, including both large and moderate effects resulting in an imprecision of estimates.

Little evidence was found in secondary outcomes assessed on participants undergoing knee or hip arthroplasty, suggesting that PrAT may reduce pain and risk of fall before surgery, and pain and stiffness one month after surgery, but in this case, the evidence is very uncertain.

Interventions’ heterogeneity was high in the main program body (exercise or education), delivery mode (synchronous or asynchronous), and dosage (from 20 to 3780 min). Since benefits and harms can be associated with volumes of exercise interventions, and a relationship between dosing and effect size exists [51], guidelines for exercise dosage in prehabilitation are needed to compare different intervention types and pool results of primary studies. Compliance with PrAT programs was very high, suggesting great patient engagement and motivation. Nevertheless, no information has been reported on participants’ compliance per the intensity of the performed sessions, which could affect the true effect size of the treatment.

### Comparison With Previous Studies

Literature on PrAT in musculoskeletal diseases is underdeveloped and mainly focused on knee and hip, with little evidence of spine surgery. Despite the lack of evidence, prehabilitation is gaining relevance in orthopedic surgery [52] and, in other fields of medicine (eg, major surgery or metabolic dysfunctions), yet is widely delivered through advanced technology systems [53,54] to properly meet the care needs of the target population, encourage adherence, and facilitate monitoring, with similar findings. Several systematic reviews exist assessing the effects of traditional prehabilitation programs for patients undergoing joint replacement [9,11,55], but they mostly focussed on the postoperative intervention effect.

### Clinical and Research Implications

Given that poor function and high preoperative pain levels are associated with worse postoperative outcomes and higher length of stay [11,16,56], PrAT could be an important intervention for patients presenting with these preoperative clinical characteristics. The high compliance observed in the included studies indicates strong patient engagement, which supports the feasibility of integrating PrAT into preoperative protocols. However, the variability in intervention types and dosages underscores the need for guidelines on exercise volume and intensity to optimize outcomes in prehabilitation interventions. Indeed, in our systematic review, the interventions could include low-impact warm-up, mobility, strengthening, proprioceptive exercises, and cool-down [44,50] or less structured interventions such as exercise suggestions, walking, stretching, and yoga practicing [45-49]. Exercise administration has proved to be effective in improving outcomes [16]. Thus, also educating patients to exercise properly to improve their conditioning before surgery could be a straightforward and effective strategy. However, to investigate its effectiveness, overall and of the individual components of multimodal interventions, it is necessary to collect data on what participants actually did after receiving education, such as through prehabilitation diaries. This information was not collected in any of the studies where prehabilitation included an educational component.

From a research perspective, further rigorous and well-designed primary studies are needed to provide strong evidence on the effects dimension provided by PrAT, both before and after surgery, extending the investigation to a wider range of musculoskeletal diseases. Additionally, monitoring patient compliance, per attendance and session intensity, and accurately reporting these data, will be crucial for understanding the true impact of PrAT. Developing standardized protocols and robust evidence will ultimately guide clinical practice and enhance the prehabilitation of patients with musculoskeletal issues awaiting surgery.

### Strengths and Limitations

To the best of our knowledge, this study has the strength of being the first systematic review assessing the effectiveness of PrAT for patients waiting for musculoskeletal surgery. It also analyzed the effect of intervention at pre and postoperative time points, collecting data from trials providing prehabilitation which included a component of exercise. However, limitations could be assessed at both review and study levels. At the review level, we did not assess grey literature and we planned GRADE assessment only for the primary outcome, as a proxy of clinical importance, because we expected that a patient undergoing PrAT could mainly improve their function during motor tasks [9,11]. Additionally, all the meta-analyses included a small number of studies. While this is methodologically reasonable, readers should consider it when interpreting the robustness of the results. Another limitation is that we did not consider overall WOMAC function but the dimensions presented in the WOMAC scale, considering function as primary outcome and pain and stiffness as secondary outcomes.

At the study level, we found high heterogeneity of the delivered programs, including exercise as the main body of prehabilitation [50], in equal shares with education [44], or as a very marginal part in the form of instructions or suggestions [45,48]. Moreover, the modality of exercise delivery (instructions or suggestions) does not allow for objective verification of what participants actively did, if they had, to perform physical exercise as intended. Indeed, collecting information on the compliance related to the “educational contents” vision or reading does not give any information about whether patients had applied what was suggested. Comparators’ heterogeneity should also be considered when interpreting the results because we pooled together studies assessing PrAT compared to different types of standard care.

### Conclusion

PrAT may be more effective than standard care in improving function both pre- and postoperatively, in candidates for knee or hip replacement. Little and uncertain evidence was found in secondary outcomes, with some trend of positive findings except for pain after surgery.

No quantitative results could be achieved on spine surgery candidates or for other musculoskeletal diseases. Intervention heterogeneity was high and adherence was often underassessed.

## Appendix

Multimedia Appendix 1 Additional materials.

## Abbreviations

GRADE: Grading of Recommendations Assessment, Development and Evaluation
NOS: Newcastle Ottawa Scale
NRIS: nonrandomized intervention study
PrAT: prehabilitation with advanced technologies
PRISMA: Preferred Reporting Items for Systematic Reviews and Meta-Analyses
RCT: randomized controlled trial
RoB: risk of bias
TIDier: Template for Intervention Description and Replication
TKA: total knee arthroplasty
VAS-BP: visual analogue scale for back pain
WOMAC: Western Ontario and McMaster Universities Osteoarthritis Index
