# Supplementary material for: Effects of Prehabilitation With Advanced Technologies in Patients With Musculoskeletal Diseases Waiting for Surgery: Systematic Review and Meta-Analysis
Source: J Med Internet Res. 2024 Dec 12;26:e52943. doi: 10.2196/52943 (PMC11671784; doi:10.2196/52943)
Supplement: Multimedia Appendix 1 [file jmir_v26i1e52943_app1.docx]

[Section S1 1](#_Toc138946875)

[Section S2. eMethods 5](#_Toc138946876)

[**Outcomes prioritization** 7](#_Toc138946877)

[**TIDieR checklist** 7](#_Toc138946878)

[**Cochrane Risk of bias** 7](#_Toc138946879)

[**NOS** 7](#_Toc138946880)

[Section S3. Results 9](#_Toc138946881)

[**References of included studies** 9](#_Toc138946882)

[**Studies characteristics** 9](#_Toc138946883)

[**Table S1.** TIDieR scores details for the assessment of intervention reporting of the 7 studies included in the systematic review. 10](#_Toc138946884)

[**Table S2.** Most reported outcomes. 10](#_Toc138946885)

[**Table S3.** Newcastle Ottawa Scale quality assessment for the 2 non randomized trials included in the systematic review. 11](#_Toc138946886)

[Section S4. Comparison between prehabilitation with advanced technologies vs standard care 12](#_Toc138946887)

[**Figure S3.** Forest plot of outcome pain 1, 3 and 12 months after surgery. 12](#_Toc138946888)

[Section S5. GRADE 13](#_Toc138946889)

[**Table S1**. Prehabilitation through advanced technologies compared to standard care for patients with musculoskeletal diseases waiting for surgery 13](#_Toc138946890)

# **Section S1**

**PRISMA checklist**

| **Section and Topic** | **Item #** | **Checklist item** | **Location where item is reported** |
| --- | --- | --- | --- |
| **TITLE** | | |  |
| Title | 1 | Identify the report as a systematic review. | 1 |
| **ABSTRACT** | | |  |
| Abstract | 2 | See the PRISMA 2020 for Abstracts checklist. | 2 |
| **INTRODUCTION** | | |  |
| Rationale | 3 | Describe the rationale for the review in the context of existing knowledge. | 3 |
| Objectives | 4 | Provide an explicit statement of the objective(s) or question(s) the review addresses. | 3 |
| **METHODS** | | |  |
| Eligibility criteria | 5 | Specify the inclusion and exclusion criteria for the review and how studies were grouped for the syntheses. | 4 |
| Information sources | 6 | Specify all databases, registers, websites, organisations, reference lists and other sources searched or consulted to identify studies. Specify the date when each source was last searched or consulted. | 4 |
| Search strategy | 7 | Present the full search strategies for all databases, registers and websites, including any filters and limits used. | 4 and Supplementary (Supplement 2) |
| Selection process | 8 | Specify the methods used to decide whether a study met the inclusion criteria of the review, including how many reviewers screened each record and each report retrieved, whether they worked independently, and if applicable, details of automation tools used in the process. | 4 |
| Data collection process | 9 | Specify the methods used to collect data from reports, including how many reviewers collected data from each report, whether they worked independently, any processes for obtaining or confirming data from study investigators, and if applicable, details of automation tools used in the process. | 4 |
| Data items | 10a | List and define all outcomes for which data were sought. Specify whether all results that were compatible with each outcome domain in each study were sought (e.g. for all measures, time points, analyses), and if not, the methods used to decide which results to collect. | 4 |
|  | 10b | List and define all other variables for which data were sought (e.g. participant and intervention characteristics, funding sources). Describe any assumptions made about any missing or unclear information. | 4 |
| Study risk of bias assessment | 11 | Specify the methods used to assess risk of bias in the included studies, including details of the tool(s) used, how many reviewers assessed each study and whether they worked independently, and if applicable, details of automation tools used in the process. | 4 |
| Effect measures | 12 | Specify for each outcome the effect measure(s) (e.g. risk ratio, mean difference) used in the synthesis or presentation of results. | 5 |
| Synthesis methods | 13a | Describe the processes used to decide which studies were eligible for each synthesis (e.g. tabulating the study intervention characteristics and comparing against the planned groups for each synthesis (item #5)). | 5 |
|  | 13b | Describe any methods required to prepare the data for presentation or synthesis, such as handling of missing summary statistics, or data conversions. | - |
|  | 13c | Describe any methods used to tabulate or visually display results of individual studies and syntheses. | - |
|  | 13d | Describe any methods used to synthesize results and provide a rationale for the choice(s). If meta-analysis was performed, describe the model(s), method(s) to identify the presence and extent of statistical heterogeneity, and software package(s) used. | 5 |
|  | 13e | Describe any methods used to explore possible causes of heterogeneity among study results (e.g. subgroup analysis, meta-regression). | 5 |
|  | 13f | Describe any sensitivity analyses conducted to assess robustness of the synthesized results. | - |
| Reporting bias assessment | 14 | Describe any methods used to assess risk of bias due to missing results in a synthesis (arising from reporting biases). | - |
| Certainty assessment | 15 | Describe any methods used to assess certainty (or confidence) in the body of evidence for an outcome. | 5 |
| **RESULTS** | | |  |
| Study selection | 16a | Describe the results of the search and selection process, from the number of records identified in the search to the number of studies included in the review, ideally using a flow diagram. | 5 |
|  | 16b | Cite studies that might appear to meet the inclusion criteria, but which were excluded, and explain why they were excluded. | - |
| Study characteristics | 17 | Cite each included study and present its characteristics. | 6 and Table 1 |
| Risk of bias in studies | 18 | Present assessments of risk of bias for each included study. | 8-9 |
| Results of individual studies | 19 | For all outcomes, present, for each study: (a) summary statistics for each group (where appropriate) and (b) an effect estimate and its precision (e.g. confidence/credible interval), ideally using structured tables or plots. | - |
| Results of syntheses | 20a | For each synthesis, briefly summarise the characteristics and risk of bias among contributing studies. | 9 |
|  | 20b | Present results of all statistical syntheses conducted. If meta-analysis was done, present for each the summary estimate and its precision (e.g. confidence/credible interval) and measures of statistical heterogeneity. If comparing groups, describe the direction of the effect. | 9-10-11 |
|  | 20c | Present results of all investigations of possible causes of heterogeneity among study results. | 9-10-11 |
|  | 20d | Present results of all sensitivity analyses conducted to assess the robustness of the synthesized results. | - |
| Reporting biases | 21 | Present assessments of risk of bias due to missing results (arising from reporting biases) for each synthesis assessed. | - |
| Certainty of evidence | 22 | Present assessments of certainty (or confidence) in the body of evidence for each outcome assessed. | Supplementary (Supplement 5) |
| **DISCUSSION** | | |  |
| Discussion | 23a | Provide a general interpretation of the results in the context of other evidence. | 11 |
|  | 23b | Discuss any limitations of the evidence included in the review. | 13 |
|  | 23c | Discuss any limitations of the review processes used. | 13 |
|  | 23d | Discuss implications of the results for practice, policy, and future research. | 12-13 |
| **OTHER INFORMATION** | | |  |
| Registration and protocol | 24a | Provide registration information for the review, including register name and registration number, or state that the review was not registered. | 1 |
|  | 24b | Indicate where the review protocol can be accessed, or state that a protocol was not prepared. | 1 |
|  | 24c | Describe and explain any amendments to information provided at registration or in the protocol. | - |
| Support | 25 | Describe sources of financial or non-financial support for the review, and the role of the funders or sponsors in the review. | 14 |
| Competing interests | 26 | Declare any competing interests of review authors. | 14 |
| Availability of data, code and other materials | 27 | Report which of the following are publicly available and where they can be found: template data collection forms; data extracted from included studies; data used for all analyses; analytic code; any other materials used in the review. | 14 |

**Differences between study protocol and publication**

According to the bio-psycho-social definition proposed by the International Classification of Functioning, Disability and Health (ICF), “functioning” refers to physical structures integrity, physiology, activity and participation (1), therefore we considered all the dimensions evaluated by the WOMAC scale. Thus, the function subscale was adopted as primary outcome, while pain and stiffness as secondary outcome.

# **Section S2. eMethods**

1. **Inclusion criteria**

In particular, according to the PICOS (Population, Intervention, Comparison, Outcomes, Study type) format, trials were included if they met the following criteria: 1) participants from 18 years old, of both sexes, affected by any musculoskeletal disease or dysfunction (as defined by the International Classification of Diseases (ICD-9) through the following codes (2): 710–721, 723.1, 724, 725–729, 730.3, 730.9, 731–733, 734–736, 737, 738, 739); 2) the intervention was a prehabilitation program delivered using advanced technologies (the administrated intervention program has to include physical exercise in the form of synchronous/asynchronous sessions, education to autonomous exercise execution or activities suggestion); 3) the comparison was standard care (as defined by the authors of the included studies). No limitations have been defined for the prehabilitation program duration. Only papers in English language have been considered.

1. **PICO QUESTION**

P=musculoskeletal disease or dysfunction, as defined by the International Classification of Disease (ICD-9);

I=prehabilitation delivered with advanced technologies, including exercise and education;

C=usual care or standard counseling, as defined by the author;

O=function, pain, strength, risk of fall, autonomy, satisfaction, quality of life, adherence to treatment, adverse events.

**SEARCH STRATEGY**

**PubMed**

("preoperative"[tiab] OR "pre-operative"[tiab] OR "Preoperative Exercise"[Mesh] OR "Preoperative Exercises" [tiab] OR "Pre operative Conditioning" [tiab] OR "Pre operative Rehabilitation" [tiab] OR "Preoperative Rehabilitation" [tiab] OR "Preoperative Conditioning" [tiab] OR "Pre-operative Exercise" [tiab] OR "Pre operative Exercise" [tiab] OR "Pre-operative Exercises" [tiab] OR "Prehabilitation" [tiab]) AND ("Telerehabilitation"[Mesh] OR "telerehabilitation"[tiab] OR "tele-rehabilitation"[tiab] OR "tele rehabilitation"[tiab] OR "virtual rehabilitation"[tiab] OR "virtual"[tiab] OR "Remote Consultation"[Mesh] OR "remote consultation" [tiab] OR "Videoconferencing"[Mesh] OR "Wearable Electronic Devices"[Mesh] OR "activity sensor*" [tiab] OR "advanced technolog*" [tiab] OR "technology system*" [tiab] OR "health technolog*" [tiab] OR "biomedical technolog*" [tiab] OR "App" [tiab] OR "mobile application*" [tiab] OR "mobile health" [tiab] OR "visual feedback" [tiab] OR "Remote" [tiab] OR "home-based" [tiab] OR "web-based" [tiab] OR "phone-based" [tiab] OR "internet-based" [tiab] OR "online" [tiab]) AND ("Orthopedics"[Mesh] OR "musculoskeletal system"[MeSH Terms] OR Musculoskeletal [tiab]) AND ((clinical[Title/Abstract] AND trial[Title/Abstract]) OR clinical trials as topic[MeSH Terms] OR clinical trial[Publication Type] OR random*[Title/Abstract] OR random allocation[MeSH Terms] OR therapeutic use[MeSH Subheading])

**EMBASE**

('preoperative treatment'/exp OR 'preoperative treatment' OR 'preoperative exercise'/exp OR 'preoperative exercise' OR prehabilitation) AND (online OR virtual OR 'telerehabilitation'/exp OR 'virtual reality'/exp OR 'virtual reality system'/exp OR 'sensor'/exp OR sensor OR 'telemonitoring'/exp OR 'telemonitoring' OR 'teleconsultation'/exp OR 'teleconsultation' OR 'web-based intervention'/exp OR 'web-based intervention') AND ('orthopedic surgery'/exp OR 'orthopedic surgery' OR 'musculoskeletal disease'/exp OR 'musculoskeletal disease' OR 'musculoskeletal system'/exp OR 'musculoskeletal system')

**CINHAL**

(Prehabilitation OR “preoperative rehabilitation” AND Musculoskeletal)

**PEDro**

(“Prehabilitation” AND Musculoskeletal AND Clinical trial) AND (Preoperative rehabilitation AND Orthopaedic AND Clinical trial)

**COCHRANE LIBRARY**

("preoperative" OR "pre-operative" OR "preoperative exercise" OR "preoperative exercises" OR "pre operative conditioning" OR "pre operative rehabilitation" OR "preoperative rehabilitation" OR "preoperative conditioning" OR "pre operative exercise" OR “pre operative exercise” OR “pre-operative exercise” OR "prehabilitation") AND ("Telerehabilitation" OR "telerehabilitation" OR "tele-rehabilitation" OR "tele rehabilitation" OR "virtual rehabilitation" OR "virtual" OR "Remote Consultation" OR "Videoconferencing" OR "Wearable Electronic Devices" OR "activity sensor*" OR "advanced technolog*" OR "technology system*" OR "health technolog*" OR "biomedical technolog*" OR "App" OR "mobile application*" OR "mobile health" OR "visual feedback" OR "Remote" OR "home-based" OR "web-based" OR "phone-based" OR "internet-based" OR "online") AND (Orthopedics OR "musculoskeletal system" OR Musculoskeletal

No restriction for publication year was applied.

1. **Data extraction**

## **Outcomes prioritization**

When trialists reported outcome data for more than one function scale, we extracted data on the scale that was highest on the following a priori defined list: (i) Western Ontario McMaster Osteoarthritis Index (WOMAC function subscale), (ii) Knee Osteoarthritis Outcome Score (KOOS function daily living), (iii) Lower Extremity Functional Scale (LEFS), (iv) Chair Stand Test and Short Physical Performance Battery (SPPB).

## **TIDieR checklist**

For interventions, we applied the reporting checklist of 12-items TIDieR checklist (name, purpose, materials, procedure, who provided the intervention, how, where, when and how much, modifications, tailoring and adherence) extracting the supporting information (3). For each item, the intervention reporting quality score was calculated using a 3-points Likert scale (0 = not reported, 1 = partially reported, 2 = adequately reported) and, for each paper, the total score was calculated.

1. **Risk of bias and methodological quality**

## **Cochrane Risk of bias**

In the RoB tool, we assessed the following five domains: randomization process, allocation concealment, blinding of participants and personnel, blinding of outcomes assessment, incomplete outcome data and selective reporting. For each domain, the judgement could be “low risk”, “high risk” or “unclear risk” of bias, and the overall study bias was rated as “low risk of bias” if all domains were classified as “low risk”, “unclear risk of bias” if there was at least one domain rated as “unclear risk” and “high risk of bias” if there was at least one domain rated as “high risk”.

## **NOS**

In the NOS, we assessed for each study the domains of selection (representativeness of intervention cohort, selection of non-intervention cohort, ascertainment of intervention, demonstration that outcomes were not present at the start of the study), comparability (comparability of cohorts on basis of design or analysis) and outcome (assessment of outcome, adequacy of follow up length for outcomes to occur and for cohort). The overall quality score was given by summing up the number of “stars” attributed to each of the three domains, according to the methodological quality (maximum of four stars for “selection”, two for “comparability” and three for “outcome”. A score of 6 or higher was judged as “Good quality”; a score between 5 and 3 was considered “Fair quality”; 2 or less was judged as “Poor quality” (4).

1. **GRADE**

Five GRADE domains—study limitations, inconsistency, imprecision, indirectness, and publication bias—were analytically assessed.

For the domain ‘study limitation’ we assessed the risk of bias according to study design. For RCTs we used the selection and detection bias as proxy judgement for low, high or unclear risk of bias. For ‘inconsistency’ we looked at the I2 considering high heterogeneity when >75%. For ‘imprecision’, we used the guiding principles of thresholds for small (SMD = ±0.2), moderate (SMD = ±0.5), and large effects (SMD = ±0.8) in order to establish thresholds using a partially contextualized approach (5–7). Details of downgrade rules are reported in Supplement 5.

We started from RCTs, thus “high certainty of evidence”. We assessed domains that can downgrade this level. Specifically:

Reasons for downgrading

**Risk of bias:**

**-1** high or unclear risk of selection bias OR outcome reporting bias (RoB); total score of 4 to 6 at the NOS;

**-2** high or unclear risk of selection bias AND outcome reporting bias (RoB); total score of 0 to 3 at the NOS.

**Imprecision:**

We used the partially contextualized approach on the primary outcome (magnitude of desirable effects is judged as trivial or none, small, moderate, or large). Thus, we following steps:

- Step 1. Define your outcome as dichotomous or continuous. Our primary outcome was function assessed as continuous outcome.
- Step 2. Set thresholds for absolute effects of health outcomes that correspond to small, moderate or large effects, both desirable and undesirable (note, that outcomes, e.g., mortality, can be a desirable outcome if it is reduced or undesirable if it is increased). In terms of absolute value, the boundaries will be the same regardless of desirability, such that trial effect coorespond to 0.0 < |SMD| ≤ 0.2, small effects correspond to 0.2 < |SMD| ≤ 0.5, moderate effects correspond to 0.5 < |SMD| ≤ 0.8, and large effects correspond to |SMD| > 0.8. If more than one threshold is crossed one would rate down for imprecision by up to three levels considering the impact of different population baseline risks(1).

These SMDs were back-translated to obtain the corresponding thresholds on a typical scale (i.e., WOMAC subscales for function, pain and stiffness assessment, TUG test for risk of fall assessment) by multiplying the SMD by a typical among-person SD (e.g. the SD of the control group at baseline from the most representative high-quality trial) (38).Thus, moving from the baseline SD values of the control group of the most representative high-quality trial (An 2021), we obtain the following translated thresholds:

- - 1.3, 3.5 and 5.7 points of WOMAC Function, for small, moderate and large effect respectively (SD used 7.1);
  - 0.3, 0.9 and 1.4 points of WOMAC Pain, for small, moderate and large effect respectively (SD used 1.7);
  - 0.2, 0.5 and 0.7 points of WOMAC Stiffness, for small, moderate and large effect respectively (SD used 0.9);
  - 0.4, 0.9 and 1.5 seconds of TUG test, for small, moderate and large effect respectively (SD used 1.85).
- Step 3. Choose the target of the rating of certainty of evidence in relation to those thresholds for the point estimate of the absolute effect. That is decide if you rate if the effect lies between two thresholds (i.e., between small desirable and undesirable effects or between small and moderate or moderate and large) or beyond the threshold for large effects or decide if you rate the certainty that the effect is beyond or below one of the thresholds.
- Step 4. Calculate the absolute effect estimates for the body of evidence for the outcome of interest including its confidence intervals based on the baseline risk and relative effect or if relevant the meta-analytic estimate of the risk difference across studies (e.g., if there are very small number of events in the studies).
- Step 5. Determine how many thresholds the confidence interval crosses regardless of if the effect estimates suggest a desirable or undesirable health effect (do not count the “no effect” as a threshold).
- Step 6. Rate down by as many levels as thresholds are crossed.
- Step 7. If the effect is large (i.e., the point estimate falls beyond the threshold for large effects) and if it is based on an apparently small number of events or participants, consider using the review information size (RIS) by calculating the required sample size for small, moderate or large effects for the outcome of interest to determine if further rating down is required because the RIS is not met (see calculator in this article). Otherwise, use the rating in step 6. If the effect appears to be trivial or none (i.e., the point estimate falls within the threshold of trivial or no effect to small desirable and undesirable effects), check if the RIS for trivial to no effects is met, for example, for the assessment of equivalence of interventions, to determine if further rating down is required. Otherwise, use the rating in step 5.

We used the the instructions and example published on Schünemann 2022 on  their [Appendix 2a and b](https://www.sciencedirect.com/science/article/pii/S0895435622001883?via%3Dihub#appsec1)): <https://www.gradepro.org/calc/reviewinformationsize>.

**Indirectness:**

We did not downgrade for indirectness since we reached the target population of interest. For ‘indirectness’ we referred to the review PICO question. For ‘publication bias’ if more than 10 studies were found we inspected it by the funnel plot The overall mean differences, at both preoperative and postoperative time points, fall over the MD threshold suggesting a large effect of the intervention, while the interval of confidence is included in the “moderate effect” range of MD values.

**Inconsistency:**

**-1** for statistical inconsistecy I^2^ >75% or methodological inconsistency (e.g., RCT and observational studies pooled together)

**-2** for statistical inconsistecy I^2^ >90%

# **Section S3. Results**

## **References of included studies**

Culliton, S. E., Bryant, D. M., MacDonald, S. J., Hibbert, K. M., & Chesworth, B. M. (2018). Effect of an e-learning tool on expectations and satisfaction following total knee arthroplasty: a randomized controlled trial. *The Journal of Arthroplasty*, *33*(7), 2153-2158.

Juneyoung, L. Y., Porucznik, C. A., Gren, L. H., Guan, J., Joyce, E., Brodke, D. S., ... & Bisson, E. F. (2019). The impact of preoperative mindfulness-based stress reduction on postoperative patient-reported pain, disability, quality of life, and prescription opioid use in lumbar spine degenerative disease: a pilot study. *World neurosurgery*, *121*, e786-e791.

Chavez, J. L., Porucznik, C. A., Gren, L. H., Guan, J., Joyce, E., Brodke, D. S., ... & Bisson, E. F. (2020). The impact of preoperative mindfulness-based stress reduction on postoperative outcomes in lumbar spine degenerative disease: 3-month and 12-month results of a pilot study. *World Neurosurgery*, *139*, e230-e236.

Yin, B., Goldsmith, L., & Gambardella, R. (2015). Web-based education prior to knee arthroscopy enhances informed consent and patient knowledge recall: a prospective, randomized controlled study. *JBJS*, *97*(12), 964-971.

Soeters, R., White, P. B., Murray-Weir, M., Koltsov, J. C., Alexiades, M. M., & Ranawat, A. S. (2018). Preoperative physical therapy education reduces time to meet functional milestones after total joint arthroplasty. *Clinical orthopaedics and related research*, *476*(1), 40.

An, J., Ryu, H. K., Lyu, S. J., Yi, H. J., & Lee, B. H. (2021). Effects of preoperative telerehabilitation on muscle strength, range of motion, and functional outcomes in candidates for total knee arthroplasty: a single-blind randomized controlled trial. *International Journal of Environmental Research and Public Health*, *18*(11), 6071.

Doiron-Cadrin, P., Kairy, D., Vendittoli, P. A., Lowry, V., Poitras, S., & Desmeules, F. (2020). Feasibility and preliminary effects of a tele-prehabilitation program and an in-person prehablitation program compared to usual care for total hip or knee arthroplasty candidates: a pilot randomized controlled trial. *Disability and rehabilitation*, *42*(7), 989-998.

## **Studies characteristics**

The participants were diagnosed with knee osteoarthritis (N=2), hip or knee osteoarthritis (N=1), primary meniscal tear (N=1) or lumbar degenerative disease (N=1). In most of the studies (N=4) the intervention was delivered using an asynchronous modality, by means of a smartphone/tablet and/or a web-page link.

## **Table S1.** TIDieR scores details for the assessment of intervention reporting of the 7 studies included in the systematic review.

| AUTHOR AND YEAR | Item 1  Brief name | Item 2  Why | Item 3  What - materials | Item 4  What - procedures | Item 5  Who provided | Item 6  How | Item 7  Where | Item 8  When and how much | Item 9  Tailoring | Item 10  Modifications | Item11  How well - Planned | Item 12  How well -Actual | TOTAL SCORE |
| --- | --- | --- | --- | --- | --- | --- | --- | --- | --- | --- | --- | --- | --- |
| Yi, 2018 | 2 | 2 | 2 | 2 | 1 | 1 | 2 | 2 | 0 | 0 | 1 | 1 | 16 |
| Chavez, 2020 | 2 | 2 | 2 | 2 | 1 | 1 | 2 | 2 | 0 | 0 | 1 | 1 | 16 |
| Yin, 2015 | 2 | 2 | 2 | 2 | 1 | 2 | 2 | 1 | 0 | 0 | 1 | 1 | 16 |
| Culliton, 2018 | 2 | 2 | 2 | 2 | 2 | 2 | 2 | 2 | 0 | 0 | 1 | 1 | 18 |
| Soeter, 2017 | 2 | 2 | 2 | 1 | 1 | 2 | 2 | 1 | 1 | 0 | 1 | 1 | 16 |
| An, 2021 | 2 | 2 | 2 | 2 | 2 | 1 | 2 | 2 | 1 | 0 | 0 | 1 | 17 |
| Doiron-Cardin, 2020 | 2 | 2 | 2 | 2 | 2 | 1 | 2 | 2 | 1 | 0 | 0 | 1 | 17 |

## **Table S2.** Most reported outcomes.

|  | Primary outcome | Secondary outcomes | | | | | | | |
| --- | --- | --- | --- | --- | --- | --- | --- | --- | --- |
|  | **Function** | **Pain** | **Stiffness** | **Risk of fall** | **Autonomy in the activities of daily living** | **Patient’s expectations, satisfaction** | **Health related quality of life** | **Adherence to treatment** | **Symptoms and activities** |
| Chavez 2020 |  | X |  |  |  |  | X | X |  |
| Yi 2018 |  | X |  |  |  |  | X | X |  |
| Yin 2015 |  |  |  |  |  | X |  | X |  |
| Culliton 2018 | X | X |  |  | X | X |  |  | X |
| Soeter 2018 | X | X | X |  | X |  |  | X |  |
| An 2021 | X | X | X | X |  |  |  |  | X |
| Doiron-Cardin 2020 | X | X |  | X |  | X |  |  | X |

## **Table S3.** Newcastle Ottawa Scale quality assessment for the 2 non randomized trials included in the systematic review.

| Author and year | 1.Selection | | | | 2.Comparability | 3.Outcome | | Overall quality score |
| --- | --- | --- | --- | --- | --- | --- | --- | --- |
|  | 1) Representativeness of the sample | 2) Sample size | 3) Non-respondents | 4) Ascertainment of the exposure (risk factor): | 1) The subjects in different outcome groups are comparable | 1) Assessment of the outcome | 2) Statistical test: |  |
| Yi 2018 | ⋆ | ⋆ |  |  |  | ⋆ |  | Poor quality |
| Chavez 2020 | ⋆ | ⋆ |  |  |  | ⋆ |  | Poor quality |

**Thresholds for converting the Newcastle-Ottawa scales to AHRQ standards**

- Good quality: 3 or 4 stars in selection domain AND 1 or 2 stars in comparability domain AND 2 or 3 stars in outcome/exposure domain
- Fair quality: 2 stars in selection domain AND 1 or 2 stars in comparability domain AND 2 or 3 stars in outcome/exposure domain
- Poor quality: 0 or 1 star in selection domain OR 0 stars in comparability domain OR 0 or 1 stars in outcome/exposure domain

# **Section S4. Comparison between prehabilitation with advanced technologies vs standard care**

**Figure S1.** Forest plot of TUG test at the pre-operative time point.


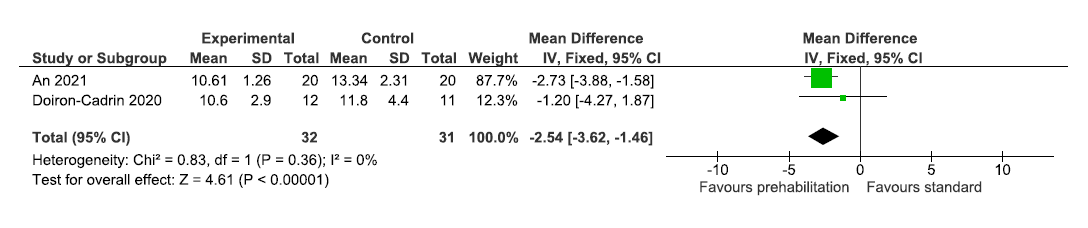


**Figure S2.** Forest plot of WOMAC stiffness subscale at the pre-operative time point and 1 month after surgery.


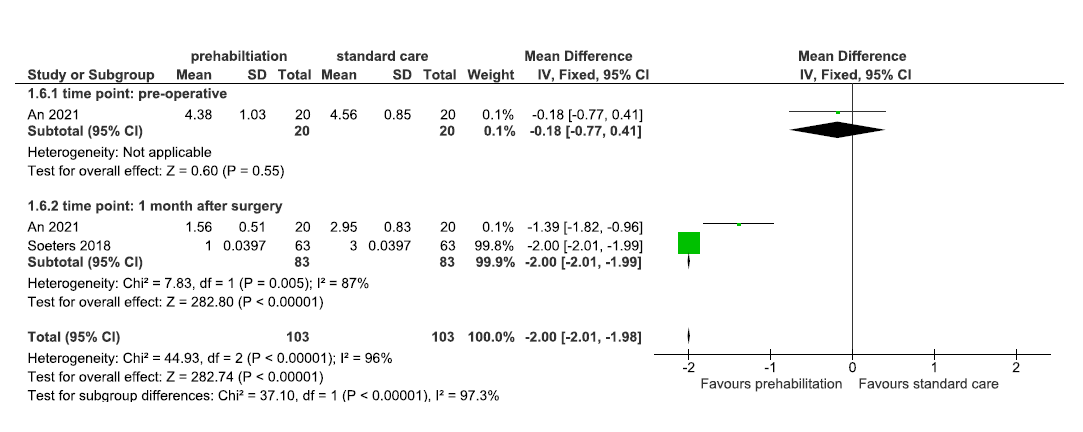


**Figure S3.** Forest plot of WOMAC pain subscale at pre-operative time point.


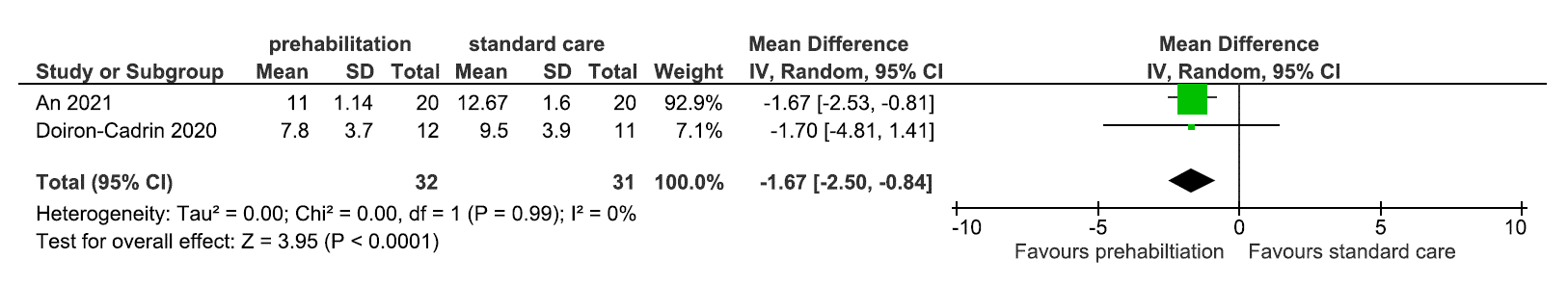


**Figure S4.** Forest plot of outcome pain 1, 3 and 12 months after surgery.


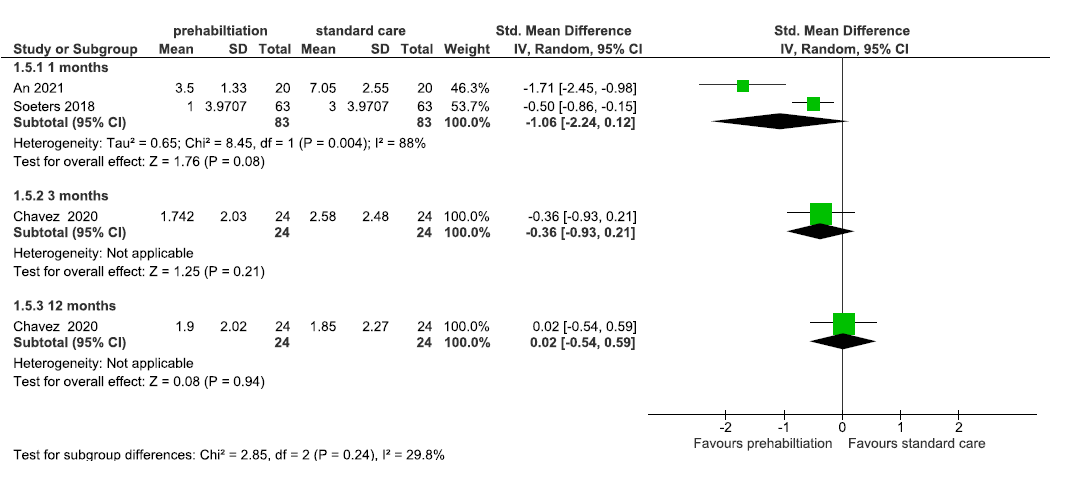


# **Section S5. GRADE**

## **Table S1**. Prehabilitation through advanced technologies compared to standard care for patients with musculoskeletal diseases waiting for surgery

| Certainty assessment | | | | | | | № of patients | | Effect | | Certainty | Importance |
| --- | --- | --- | --- | --- | --- | --- | --- | --- | --- | --- | --- | --- |
| № of studies | Study design | Risk of bias | Inconsistency | Indirectness | Imprecision | Other considerations | prehabilitation through advanced technologies | standard care | Relative (95% CI) | Absolute (95% CI) |  |  |
| function (WOMAC subscale) - time point: pre-operative | | | | | | | | | | | | |
| 2 | randomised trials | serious^a^ | not serious | not serious | serious^b^ | none | 32 | 31 | - | MD 7.59 lower (10.85 lower to 4.33 lower) | ⨁⨁◯◯ Low |  |
| function (WOMAC subscale) - time point: 1 month after surgery | | | | | | | | | | | | |
| 2 | randomised trials | serious^a^ | serious^c^ | not serious | serious^b^ | none | 83 | 83 | - | MD 7.84 lower (11.8 lower to 3.88 lower) | ⨁◯◯◯ Very low |  |
| pain (WOMAC subscale) time point: pre-operative | | | | | | | | | | | | |
| 2 | randomised trials | serious^a^ | not serious | not serious | serious^b^ | none | 32 | 31 | - | MD 1.67 lower (2.5 lower to 0.84 lower) | ⨁⨁◯◯ Low |  |
| pain - time point: 1 month after surgery | | | | | | | | | | | | |
| 2 | randomised trials | serious^a^ | serious^c^ | not serious | extremely serious^d^ | none | 107 | 107 | - | SMD 1.06 SD lower (2.24 lower to 0.12 higher) | ⨁◯◯◯ Very low |  |
| pain after surgery - 3 months | | | | | | | | | | | | |
| 1 | non-randomised studies | very serious^e^ | not serious | not serious | extremely serious^f^ | none | 24 | 24 | - | SMD 0.36 lower (0.93 lower to 0.21 higher) | ⨁◯◯◯ Very low |  |
| pain after surgery - 12 months | | | | | | | | | | | | |
| 1 | non-randomised studies | very serious^e^ | not serious | not serious | extremely serious^f^ | none | 24 | 24 | - | SMD 0.02 SD higher (0.54 lower to 0.59 higher) | ⨁◯◯◯ Very low |  |
| WOMAC stiffness - time point: 1 month after surgery | | | | | | | | | | | | |
| 2 | randomised trials | serious^a^ | serious^c^ | not serious | not serious | none | 103 | 103 | - | MD 2 lower (2.01 lower to 1.99 lower) | ⨁⨁◯◯ Low |  |
| WOMAC stiffness - time point: pre-operative | | | | | | | | | | | | |
| 1 | randomised trials | serious^a^ | not serious | not serious | extremely serious^f^ | none | 40 | 20 | - | MD 0.02 higher (0.49 lower to 0.53 higher) | ⨁◯◯◯ Very low |  |
| Risk of Fall (TUG) | | | | | | | | | | | | |
| 2 | randomised trials | serious^a^ | not serious | not serious | serious^b^ | none | 32 | 21 | - | MD 2.54 lower (3.62 lower to 1.46 lower) | ⨁⨁◯◯ Low |  |

CI: confidence interval; MD: mean difference; SMD: standardised mean difference

**Explanations**

a. high/unclear risk of selection (randomization and allocation) OR outcome reporting bias

b. CIs 95% crossed one threshold of effect

c. I2 > 75%

d. CIs 95% crossed two thresholds of effect and the no effect line

e. Poor quality (NOS)

f. CIs 95% crossed three thresholds of effect and no effect line

**Bibliography**

1. Stucki G, Cieza A, Melvin J. The international classification of functioning, disability and health: A unifying model for the conceptual description of the rehabilitation strategy. J Rehabil Med. 2007;39(4):279–85.

2. Smith E, Hoy DG, Cross M, Vos T, Naghavi M, Buchbinder R, et al. The global burden of other musculoskeletal disorders: Estimates from the Global Burden of Disease 2010 study. Ann Rheum Dis. 2014;73(8):1462–9.

3. Hoffmann TC, Glasziou PP, Boutron I, Milne R, Perera R, Moher D, et al. Better reporting of interventions: Template for intervention description and replication (TIDieR) checklist and guide. BMJ [Internet]. 2014;348(March):1–12. Available from: http://dx.doi.org/doi:10.1136/bmj.g1687

4. Welles G, Shea BJ, J O. The Newcastle–Ottawa Scale (NOS) for Assessing the Quality of Non-Randomized Studies in Meta-Analysis. 2000;

5. Guyatt GH, Oxman AD, Kunz R, Brozek J, Alonso-Coello P, Rind D, et al. GRADE guidelines 6. Rating the quality of evidence - Imprecision. J Clin Epidemiol. 2011;64(12):1283–93.

6. Cohen J. Statistical Power Analysis for the Behavioral Sciences. 1988;2nd editio. Available from: https://doi.org/10.4324/9780203771587

7. Zeng L, Brignardello-Petersen R, Hultcrantz M, Mustafa RA, Murad MH, Iorio A, et al. GRADE Guidance 34: update on rating imprecision using a minimally contextualized approach. J Clin Epidemiol. 2022;150(20):216–24.
